# An Inducible Nitric Oxide Synthase Dimerization Inhibitor Prevents the Progression of Osteoarthritis

**DOI:** 10.3389/fphar.2022.861183

**Published:** 2022-07-15

**Authors:** Shang Xian Bo, Wang Yan Jie, Cai De Chao, Ma Sai, Wang Zhe, Zhu Ya Kun, Guo Hui Hui, Wang Chen, Ma Xiao, Hu Zhong Yao, Yu Hao Ran, Zhang Ji Sen, Cheng Wen Dan

**Affiliations:** ^1^ Department of Orthopedic, The Second Affiliated Hospital of Anhui Medical University, Hefei, China; ^2^ Department of Orthopedic, Third Affiliated Hospital of Anhui Medical University, Hefei, China; ^3^ Fuyang Hospital of Anhui Medical University, Anhui, China

**Keywords:** osteoarthritis, nitric oxide, inducible nitric oxide synthase, inhibitor, extracellular matrix

## Abstract

**Objective:** Osteoarthritis (OA) is a degenerative joint disease. Excessive nitric oxide (NO) mediates the chondrocyte inflammatory response, apoptosis, and extracellular matrix (ECM) degradation during the occurrence and development of OA. NO in chondrocytes is mainly produced by inducible nitric oxide synthase (iNOS). The aim of this study was to design and synthesize an iNOS dimerization inhibitor and evaluate its effects on chondrocyte inflammation and articular cartilage injury in OA via *in vitro* and *in vivo* experiments.

**Design:** The title compound 22o was designed, synthesized, and screened based on a previous study. The effects of different concentrations (5, 10, and 20 μM) of compound 22o on chondrocyte inflammatory response and ECM anabolism or catabolism were evaluated by Western blot and real-time quantitative reverse transcription-polymerase chain reaction using the rat chondrocyte model of IL-1β-induced OA. Furthermore, different doses (40 and 80 mg/kg) of compound 22o were administered by gavage to a rat OA model induced by anterior cruciate ligament transection (ACLT), and their protective effects on the articular cartilage were evaluated by histopathology and immunohistochemistry.

**Results:** Compound 22o showed effective iNOS inhibitory activity by inhibiting the dimerization of iNOS. It inhibited the IL-1β-induced expression of cyclooxygenase-2 (COX-2) and matrix metalloproteinase 3 (MMP3) in the chondrocytes, decreased NO production, and significantly increased the expression levels of the ECM anabolic markers, aggrecan (ACAN), and collagen type II (COL2A1). Gavage with compound 22o was found to be effective in the rat OA model induced by ACLT, wherein it regulated the anabolism and catabolism and exerted a protective effect on the articular cartilage.

**Conclusions:** Compound 22o inhibited the inflammatory response and catabolism of the chondrocytes and reduced articular cartilage injury in the rat OA model, indicating its potential as a disease-modifying OA drug.

## Introduction

Osteoarthritis (OA) is the most common musculoskeletal disease and one of the main causes of disability and decline in quality of life among older people (Glyn-Jones et al., 2015; GBD 2019 Diseases and Injuries Collaborators, 2020). As an age-related joint disease, OA affects the health of 303 million people worldwide (GBD 2017 Diseases and Injuries Collaborators, 2018), and its prevalence continues to rise (Peat and Thomas, 2021). So far, most treatment strategies can only alleviate the symptoms of OA but not prevent disease progression. Joint replacement is the only option for patients with end-stage OA (Glyn-Jones et al., 2015; Peat and Thomas, 2021), and this poses a huge economic burden for both the individual and society.

Although OA is a complex disease and its pathogenesis is not fully understood, the inflammatory response certainly plays an important role in the progression of this condition (Robinson et al., 2016; Hunter and Bierma-Zeinstra, 2019). Nitric oxide (NO) is a free radical with multiple functions that are widely involved in a variety of physiological processes in the cells. However, excessive NO, which can act as an important inflammatory mediator, can affect the pathological process of OA by inducing the apoptosis of chondrocytes and maintaining the expression of proinflammatory cytokines (Chen et al., 2018; Ahmad et al., 2020). In addition, excessive NO-derived reactive oxygen species and reactive nitrogen species can lead to mitochondrial dysfunction and affect the survival of the chondrocytes (Maneiro et al., 2005). NO is mainly mediated by nitric oxide synthase (NOS) (Cinelli et al., 2020), which consists of three subtypes: inducible nitric oxide synthase (iNOS), endothelial NOS (eNOS), and neuronal NOS (nNOS). The production of NO in chondrocytes is mainly regulated by iNOS. iNOS does not always exist in the cells but is expressed only when chondrocytes are stimulated by bacterial lipopolysaccharide or proinflammatory cytokines resulting in the excessive production of NO (Sharma et al., 2007; Minhas et al., 2020), which finally leads to chondrocyte apoptosis and extracellular matrix (ECM) degradation. It has been shown that the iNOS inhibitor S-methylisothiourea can play an analgesic role by reducing NO production in the synovial fluid and delaying the progression of OA (More et al., 2013). Moreover, the selective iNOS inhibitor n-iminoethyl-L-lysine can prevent lipid peroxidation and reduce the apoptosis of OA chondrocytes, thereby delaying the progression of OA (Pelletier et al., 2000; Bentz et al., 2012). Therefore, iNOS inhibitors might be considered as potential disease-modifying OA drugs (DMOADs) in the future.

iNOS inhibitors are divided into two types: direct and indirect inhibitors. Direct inhibitors directly bind to iNOS and inhibit its activity, whereas indirect inhibitors target the NF-κB or STAT-1 signaling pathway and down-regulate iNOS (Ahmad et al., 2020). In the present study, a novel iNOS dimerization inhibitor 22o, which is a direct inhibitor, is chemically synthesized. The aim of this study was to evaluate the anti-inflammatory effect of the compound on chondrocytes and its protective effect on the articular cartilage in rats with OA. And its effects on the inflammatory response and articular cartilage injury were evaluated using a rat chondrocyte model of OA (*in vitro*) and a rat OA model (*in vivo*).

## Materials and Methods

### Synthesis of Compound 22o

The compound 22o (E)-N-(4-methylbenzyl)-2-(3,4,5-trimethoxystyryl)thieno[3,2-d]pyrimidin-4-amine (22o) was synthesized as described in a previous study (Chen et al., 2021).

### Regents

Phosphate-buffered saline (PBS) and Dulbecco’s modified Eagle’s medium (DMEM) were procured from Hyclone (United States). Type II collagenase and fetal bovine serum (FBS) were procured from Gibco (United States). 1% penicillin/streptomycin solution (100×), methyl thiazolyl tetrazolium (MTT), dimethyl sulfoxide (DMSO), NO synthase detection kit, NO detection kit, RIPA cell lysis buffer, phenylmethanesulfonyl fluoride (PMSF), and 5 × SDS-PAGE protein loading buffer were supplied by Beyotime (China). Recombinant rat IL-1β was purchased from Peprotech (United States). Antibodies against iNOS (ab178945), collagen type II (COL2A1; ab34712), matrix metalloproteinase 3 (MMP3; ab52915), cyclooxygenase-2 (COX-2; ab179800), matrix metalloproteinase 13 (MMP13; ab39012), and iNOS inhibitor 1400W (ab120165) were supplied by Abcam (United States). Antibodies against aggrecan (ACAN; DF7561), glyceraldehyde phosphate dehydrogenase (GAPDH; AF7021), Goat anti-rabbit IgG (H + L) HRP (S0001), and Goat anti-rabbit IgG (H + L) FITC (S0008) were procured from Affinity (China).

### Isolation and Culture of Chondrocytes

Primary rat chondrocytes were isolated from 5-day-old male SD rats, which were purchased from the Experimental Animal Center of Anhui Medical University. Specifically, the cartilage tissues obtained from the femoral heads of the SD rats were excised and washed three times with PBS containing 1% penicillin/streptomycin solution. Subsequently, 0.25% of type II collagenase was added and fully digested at 37°C for 6 h. The cell suspension was collected in a centrifuge tube and centrifuged at 1,200 rpm/min for 5 min. After centrifugation, the cell precipitate was resuspended in DMEM containing 10% FBS and 1% penicillin/streptomycin solution and cultured in a humidified incubator containing 5% CO_2_ at 37°C. Third-generation chondrocytes were used for subsequent experiments.

### Cell Viability Assay

Cell viability was determined using the MTT assay. Specifically, the rat chondrocytes were inoculated into 96-well plates at a density of 1 × 104 cells/well. After the adherence of the cells to the walls of the plates, they were treated with different concentrations (0, 5, 10, 20, and 40 μM) of compound 22o for 24 h. Next, 20 μL of MTT solution (5 mg/ml prepared with PBS) was added to each well, and the plates were incubated in a cell incubator at 37°C for 4 h. The culture medium was removed, and 150 μL DMSO was added to each well. Subsequently, the absorbance of each well was measured at 490 nm using a microplate reader.

### Inducible Nitric Oxide Synthase Activity Assay

The activity of iNOS was determined using a NO synthase detection kit. The rat chondrocytes were inoculated into 96-well plates at a density of 1 × 104 cells/well. After the adherence of the cells to the walls, 10 ng/ml of IL-1β was used alone or in combination with different concentrations (5, 10, and 20 μM) of compound 22o to the rat chondrocytes and incubated for 24 h. The culture medium was sucked out, 100 μL detection buffer +100 μL reaction solution (see the product manual for the solution preparation method) were added to each well, and the plates were incubated at 37°C for 30 min. Subsequently, the absorbance of each well was measured at 495 nm using a microplate reader. The relative activity of iNOS was calculated according to the given formula.

### Determination of Nitric Oxide Content

NO content was determined using a NO detection kit. The rat chondrocytes were inoculated onto 24-well plates at a density of 7 × 104 cells/well. After the adherence of the cells to the walls, 10 ng/ml of IL-1β was used alone or in combination with different concentrations (5, 10, and 20 μM) of compound 22o and incubated for 24 h. Next, 50 μL of the supernatant was removed from the 96-well plates, and 50 μL of Griess Reagent I and 50 μL Griess Reagent II were added to each well. Subsequently, the absorbance of each well was measured at 540 nm using a microplate reader. The content of NO was calculated according to the standard curve.

### Total Protein Extraction and Western Blot Analysis

The cells were lysed with RIPA cell lysis buffer containing 1% PMSF and incubated on ice for 30 min. The total lysate was collected and centrifuged at 12,000 rpm/min and 4°C for 10 min, and the supernatant (total protein) was extracted. Subsequently, 5 × SDS-PAGE protein loading buffer was added and boiled at 100°C for 10 min to denature the total protein. Protein samples were isolated using 8% or 10% SDS-PAGE and transferred to a polyvinylidene difluoride membrane (Millipore Immobilon-P, United States), which was blocked with Tris-buffered saline Tween containing 5% skimmed milk powder at room temperature for 2 h; the membranes were then incubated with primary antibodies at 4°C overnight. Incubation with secondary antibodies was performed at room temperature for 1 h. Finally, the proteins were photographed using the gel imaging system and quantified using Image-J. The expression levels of the protein were relatively quantified using GAPDH as the internal reference.

### Total RNA Extraction and Real-Time Quantitative Reverse Transcription-Polymerase Chain Reaction

Total RNA was extracted using the TRIzol reagent (Invitrogen, United States), and the purified RNA was reversely transcribed using the Servicebio^®^ RT First Strand cDNA Synthesis Kit (Servicebio, China). RT-qPCR was carried out using the 2×SYBR Green qPCR Master Mix (Servicebio, China). The relative gene expression was presented as multiple changes, with GAPDH as the internal reference, and calculated using the 2^−ΔΔCT^ method. The sequences of the primers used in this study are shown in Table 1.

**TABLE 1:** Primers used in RT-qPCR detection.

Genes	Forward (5′- 3′)	Reverse (5′- 3′)
ACAN	AGT​GAC​CCA​TCT​GCT​TAC​CCT​G	CTG​CAT​CTA​TGT​CGG​AGG​TAG​TG
COL2A1	GTG​TCA​AGG​GTC​ACA​GAG​GTT​AC	CGC​TCT​CAC​CCT​TCA​CAC​CT
MMP3	CAG​GTT​ATC​CTA​AAA​GCA​TTC​ACA​C	CCG​CTG​AAG​AAG​TAA​AGA​AAC​CC
COX-2	AAA​ACC​GTG​GTG​AAT​GTA​TGA​GC	GGT​GGG​CTG​TCA​ATC​AAA​TGT
GAPDH	CTG​GAG​AAA​CCT​GCC​AAG​TAT​G	GGT​GGA​AGA​ATG​GGA​GTT​GCT

### Induction and Treatment of the Rat Osteoarthritis Model

The OA model was induced by anterior cruciate ligament transection (ACLT) in male SD rats weighing 250–280 g. The rats were operated on after adapting to the environment in the animal room for 1 week. In brief, the rats were anesthetized with chloral hydrate, and the left knee joint was exposed using the medial parapatellar approach. Then, the ACL was excised and the knee joint was sutured layer by layer. In the sham operation group, only the knee joint was exposed, without the excision of the ACL (The mortality is less than 10 percent and state of the animals are fine during the modeling process). The rats were randomly divided into five groups as follows (six rats in each group): normal, sham-operated, and ACLT (treated with vehicle, or 40 and 80 mg/kg of compound 22o). The compound 22o was dissolved in 0.5% sodium carboxymethylcellulose solution. One week after surgery, gavage was performed once a day for 6 weeks (The structure of the compound does not change after entering the body, and the substance producing the therapeutic effect is still the original compound). The rats were sacrificed 24 h after the last gavage. The knee joints of the rats were collected and fixed with 4% paraformaldehyde (Servicebio, China) after pruning. They were decalcified in 10% ethylenediaminetetraacetic acid (Servicebio, China) solution for 2 months for subsequent experiments. The heart, liver, spleen, lungs, and kidneys of the rats were removed to evaluate the toxicity of compound 22o in the rats.

### Histological and Immunohistochemical Analyses

The decalcified rat knee tissues were collected, dehydrated, and embedded in paraffin. They were cut into 4 μm-thick sections and stained with hematoxylin and eosin (HE) (Servicebio, China), safranin O-fast green (Servicebio, China), alcian blue (Servicebio, China), and toluidine blue (Servicebio, China). Additionally, immunohistochemical staining was performed using specific antibodies against ACAN, COL2A1, MMP3, and MMP13. The heart, liver, spleen, lung, and kidney of the rats were stained only with HE.

For the immunofluorescence staining, the sections were dewaxed, rehydrated, repaired with citrate buffer (Servicebio, China), blocked with 3% H_2_O_2_ for 30 min, and then blocked with 5% bovine serum albumin (Servicebio, China) for 1 h. Subsequently, the sections were incubated with the specific antibody against COL2A1 at 4°C overnight and incubated with the corresponding secondary antibody at room temperature in the dark for 30 min on the next day. The nuclei were stained with destination access point identifier dye (Servicebio, China), and the sections were observed under a fluorescence microscope.

### Ethical Approval

This study was approved by the ethics committee of Anhui Medical University (LLSC20210373). All animal experiments were conducted in accordance with the international guidelines on the ethical use of animals, and all efforts were made to minimize the amount of pain or discomfort experienced by the animals.

### Statistical Analysis

All data are expressed as mean ± standard deviation. All data were statistically analyzed using one-way analysis of variance and Tukey’s multiple comparisons test in GraphPad Prism 8. A *p* value of <0.05 was considered statistically significant. All experiments were conducted at least three times.

## Results

### Design and Optimization

**FIGURE 1:**
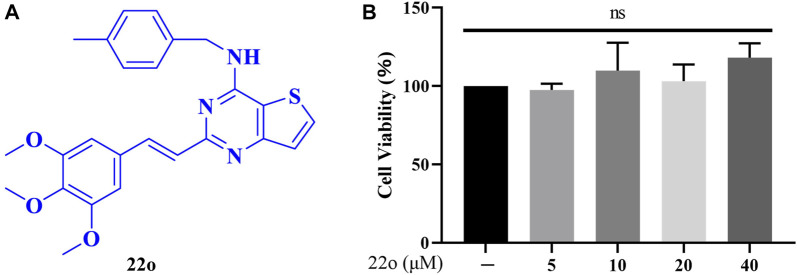
Effects of compound 22o on cell viability. **(A)** Chemical structure of compound 22o. **(B)** Rat chondrocytes were treated with compound 22o (5, 10, 20, and 40 μM) for 24 h and cell viability was assessed by MTT assay (*n* = 3). NS indicated no significance.

The analysis of a few small-molecule iNOS inhibitors showed that they contained pyrazolopyrimidine heterocyclic structures, leading us to speculate that these heterocyclic structures might constitute the structural parent nucleus of iNOS inhibitors. Besides, tetrahydrobiopterin (BH4), a critical coenzyme of iNOS, is essential for the formation of an iNOS dimer. Therefore, the binding regions of BH4 and iNOS can be used as ideal targets for the synthesis of novel iNOS inhibitors. Subsequently, through computer DOCK, it was noted that when styryl pyrazolopyrimidine heterocycle is bound to the iNOS oxygenase domain (iNOSox), it completely occupiedoccupies the binding site between BH4 and iNOSox, showing consistent orientation to that of BH4. This indicated that the styryl pyrazolopyrimidine heterocyclic parent nucleus can compete for the site of BH4 and affect the formation of an iNOS dimer. Finally, we designed and synthesized a compound library based on styryl pyrazolopyrimidine heterocycles. Based on the balance between toxicity and anti-inflammatory activity, aiming to improve the physicochemical properties and to enhance drug-like properties, we continuously optimized, screened and finally determined the title compound 22o (Figure 1A).

### Effect of Compound 22o on Cell Viability

Different concentrations of compound 22o were used to treat rat chondrocytes for 24 h to test its cytotoxicity. The cytotoxicity was determined using the MTT assay. As seen in Figure 1B, no toxic effects were observed on the chondrocytes at concentrations 5, 10, 20, and 40 μM. Therefore, the concentration gradient of 5, 10, and 20 μM was used in subsequent experiments.

### Compound 22o Inhibits Inducible Nitric Oxide Synthase Activity and Nitric Oxide Production

**FIGURE 2:**
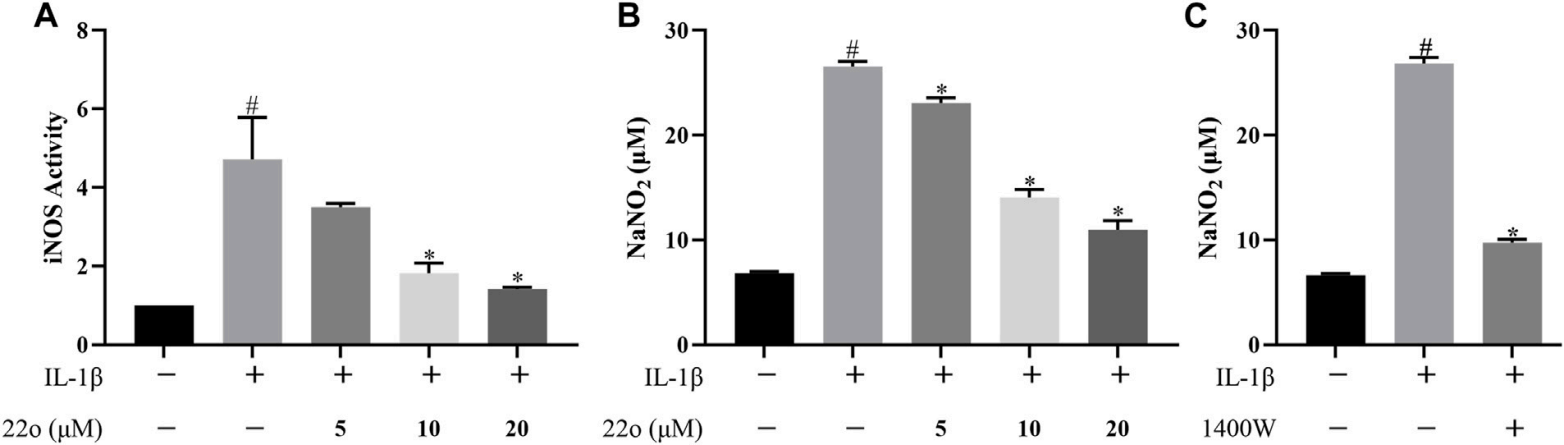
Compound 22o inhibits iNOS activity and NO production. Rat chondrocytes were treated with different concentrations of compound 22o in the presence or absence of IL-1β (10 ng/ml) for 24 h. **(A)** The activity of iNOS was evaluated by Nitric Oxide Synthase Assay Kit (*n* = 3). **(B**,**C)** Griess reagent was used to measure the NO levels in the culture supernatants (*n* = 3). #*p* < 0.05 vs. control group; *p < 0.05 vs. IL-1β group.

Rat chondrocytes were treated with IL-1β (10 ng/ml) alone or in combination with compound 22o for 24 h to investigate whether compound 22o can inhibit IL-1β-induced iNOS activity and reduce NO release by measuring iNOS activity and NO content in each group. As shown in Figure 2A, IL-1β significantly enhanced the activity of iNOS in the chondrocytes, whereas compound 22o inhibited this process at 20 μM concentration. In addition, the release of NO in the IL-1β + 20 μM compound 22o group was also significantly reduced compared to that in the IL-1β group (Figure 2B). Moreover, a selective iNOS inhibitor 1400W (100 μM) inhibited IL-1β-induced NO accumulation indicating that it was synthesized through iNOS pathway (Figure 2C). These results demonstrated that compound 22o could reduce NO release in the OA chondrocytes by inhibiting the activity of iNOS.

### Compound 22o Inhibits the Formation of the Inducible Nitric Oxide Synthase Dimer

**FIGURE 3:**
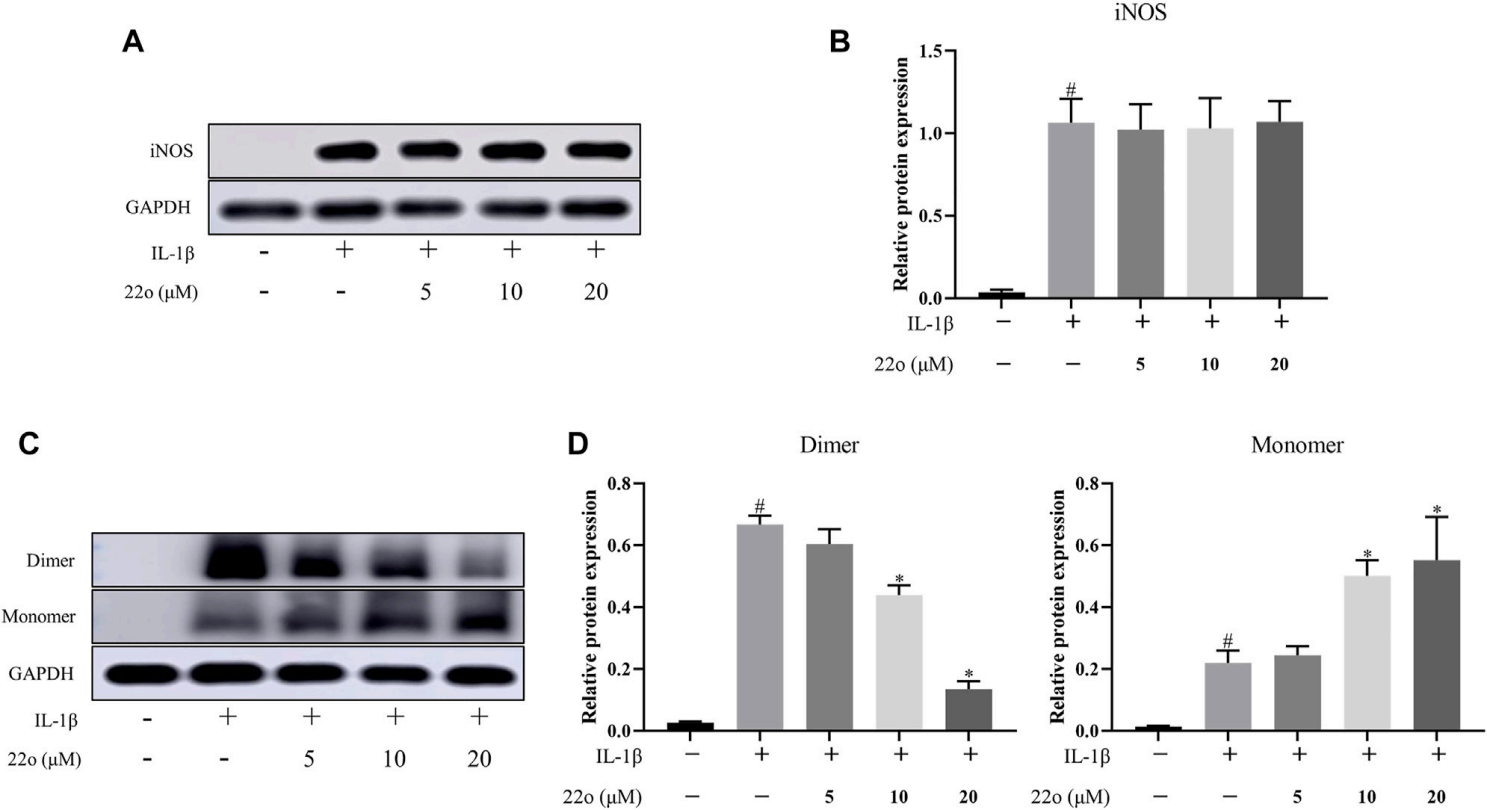
Compound 22o inhibits the formation of iNOS dimer. Rat chondrocytes were treated with different concentrations of compound 22o in the presence or absence of IL-1β (10 ng/ml) for 24 h. **(A)** Western blots and **(B)** quantitative analysis of iNOS. **(C)** Western blots and **(D)** quantitative analysis of iNOS dimer and monomer. GAPDH was employed as the internal control (*n* = 3). #*p* < 0.05 vs. control group; *p < 0.05 vs. IL-1β group.

Compound 22o directly binds to iNOS to inhibit its activity but does not affect the total iNOS protein expression (Figures 3A,B). Additionally, compound 22o is a dimerization inhibitor that inhibits the formation of the iNOS dimer. To confirm this, the effect of compound 22o on the formation of the iNOS dimer was evaluated *via* non-denaturing low-temperature gel electrophoresis. As expected, compound 22o inhibited the dimerization of the iNOS protein (Figures 3C,D). Moreover, since iNOS is active in the dimer form (Cinelli et al., 2020), compound 22o might inhibit its activity by inhibiting the formation of the iNOS dimer.

### Compound 22o Alleviates IL-1β-Induced Chondrocyte Inflammatory Response and Extracellular Matrix Degradation

**FIGURE 4:**
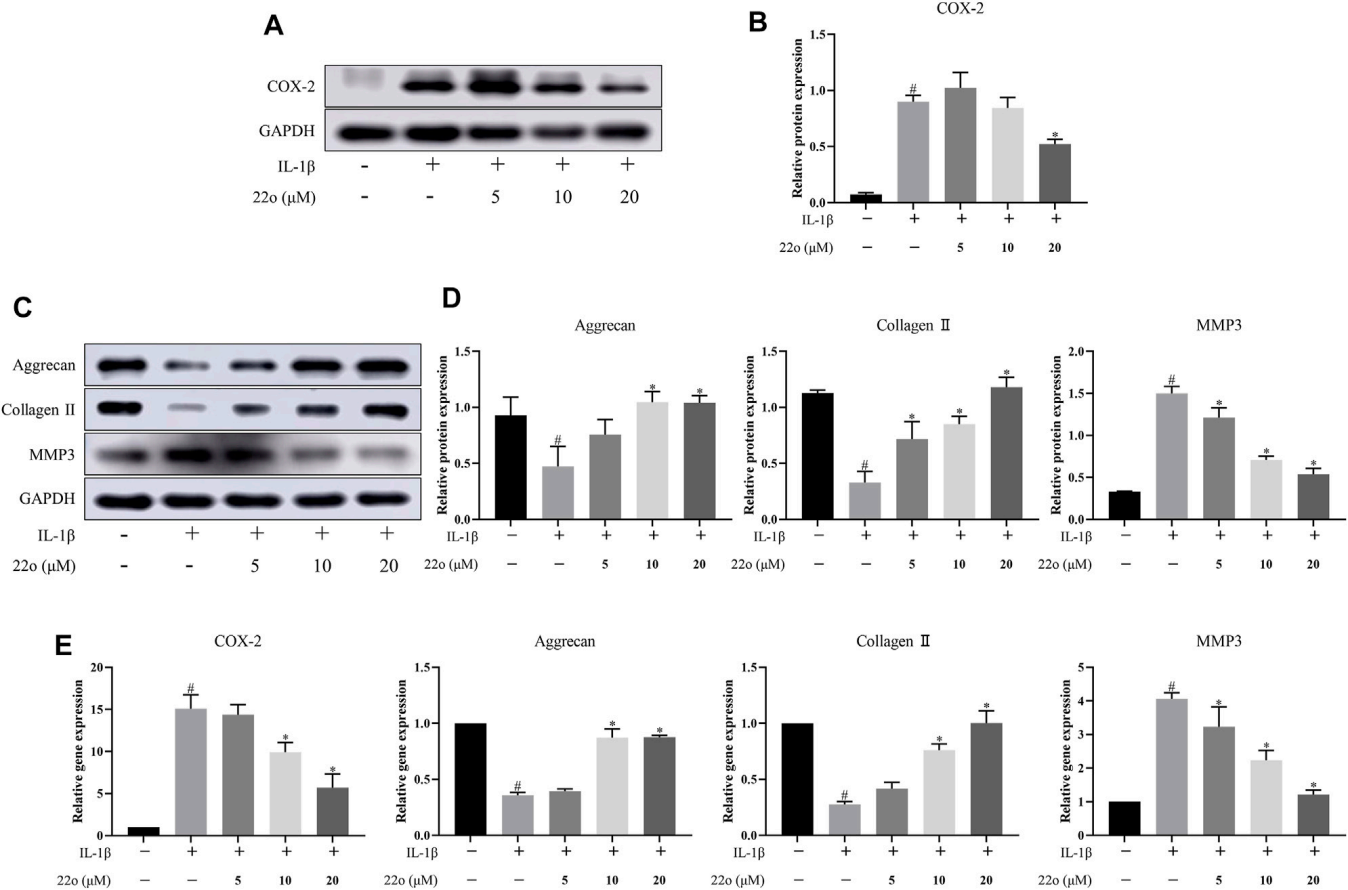
Compound 22o alleviates IL-1β-induced chondrocyte inflammatory responses and ECM degradation. Rat chondrocytes were treated with different concentrations of compound 22o in the presence or absence of IL-1β (10 ng/ml) for 24 h. **(A)** Western blots and **(B)** quantitative analysis of COX-2. **(C)** Western blots and **(D)** quantitative analysis of ACAN, COL2A1 and MMP3. GAPDH was employed as the internal control (*n* = 3). **(E)** Quantification of mRNA levels for COX-2, ACAN, COL2A1 and MMP3. Fold changes relative to control group are shown (*n* = 3). #*p* < 0.05 vs. control group; *p < 0.05 vs. IL-1β group.

Rat chondrocytes were treated with IL-1β (10 ng/ml) alone or in combination with compound 22o for 24 h to investigate whether compound 22o can improve IL-1β-induced inflammatory response and ECM degradation. The results show that treatment with 20 μM of compound 22o significantly reduced the IL-1β-induced overexpression of COX-2 (Figures 4A,B). The ACAN, COL2A1, and MMP3 were used to evaluate cartilage ECM degradation. As shown in Figure 4C, D, IL-1β significantly induced the down-regulation of ACAN and COL2A1 and up-regulation of MMP3 in the OA chondrocytes; however, treatment with different concentrations of compound 22o partially reversed this change. This was further confirmed by the RT-qPCR (Figure 4E).

### Compound 22o Alleviates the Progression of Osteoarthritis

**FIGURE 5:**
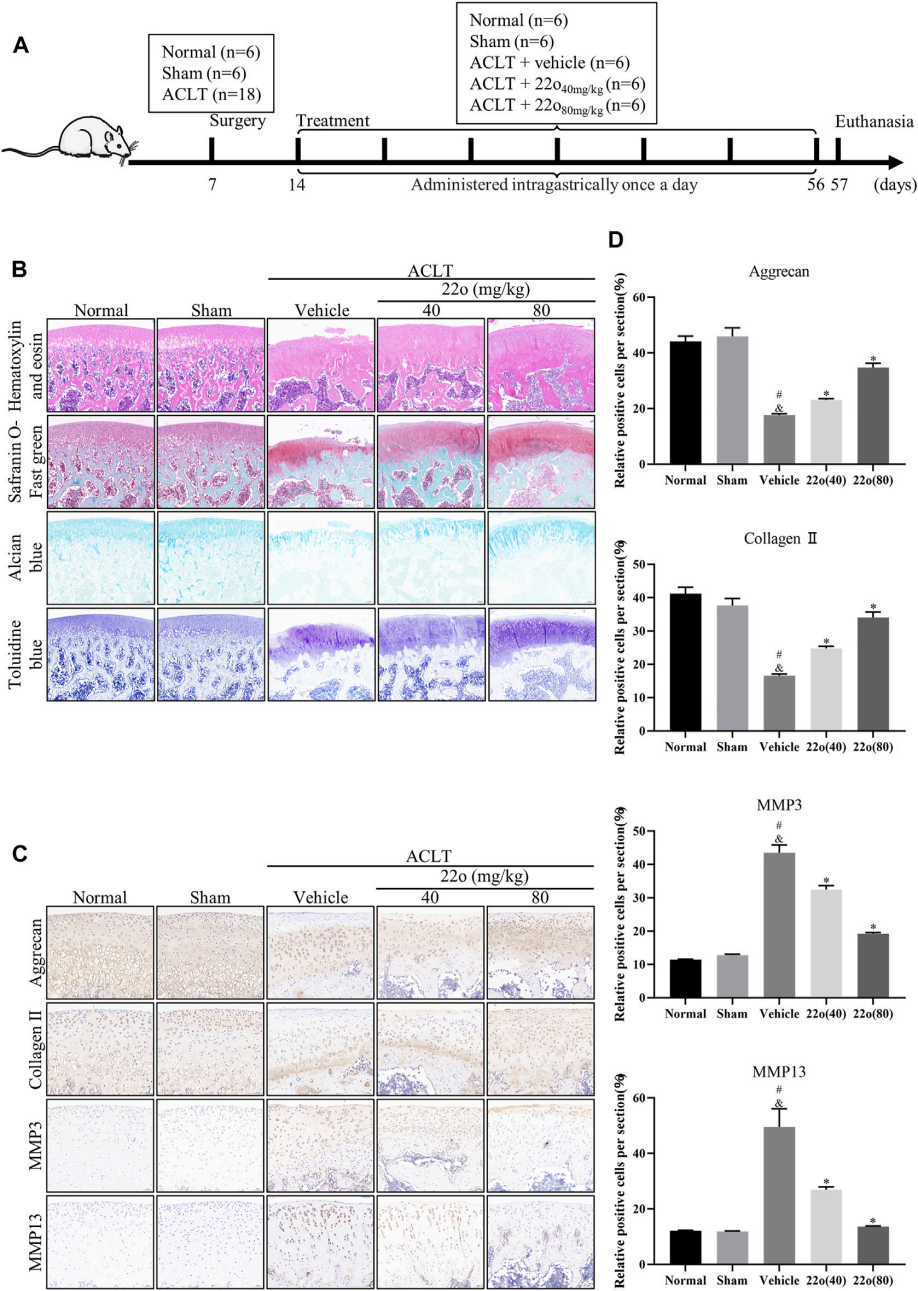
Compound 22o alleviates the progression of OA. **(A)** Schematic diagram of the animal experiment design. **(B)** Representative images of HE, safranin O-fast green, alcian blue and toluidine blue staining of cartilage samples from five groups 6 weeks after treatment (scale bars, 100 μm). **(C)** Representative images of immunohistochemistry staining and **(D)** quantitative analysis of ACAN, COL2A1 MMP3 and MMP13 in the cartilage samples from five groups 6 weeks after treatment (scale bars, 50 μm, *n* = 6). #*p* < 0.05 vs. normal group; &*p* < 0.05 vs. sham group; *p < 0.05 vs. vehicle group.

**FIGURE 6:**
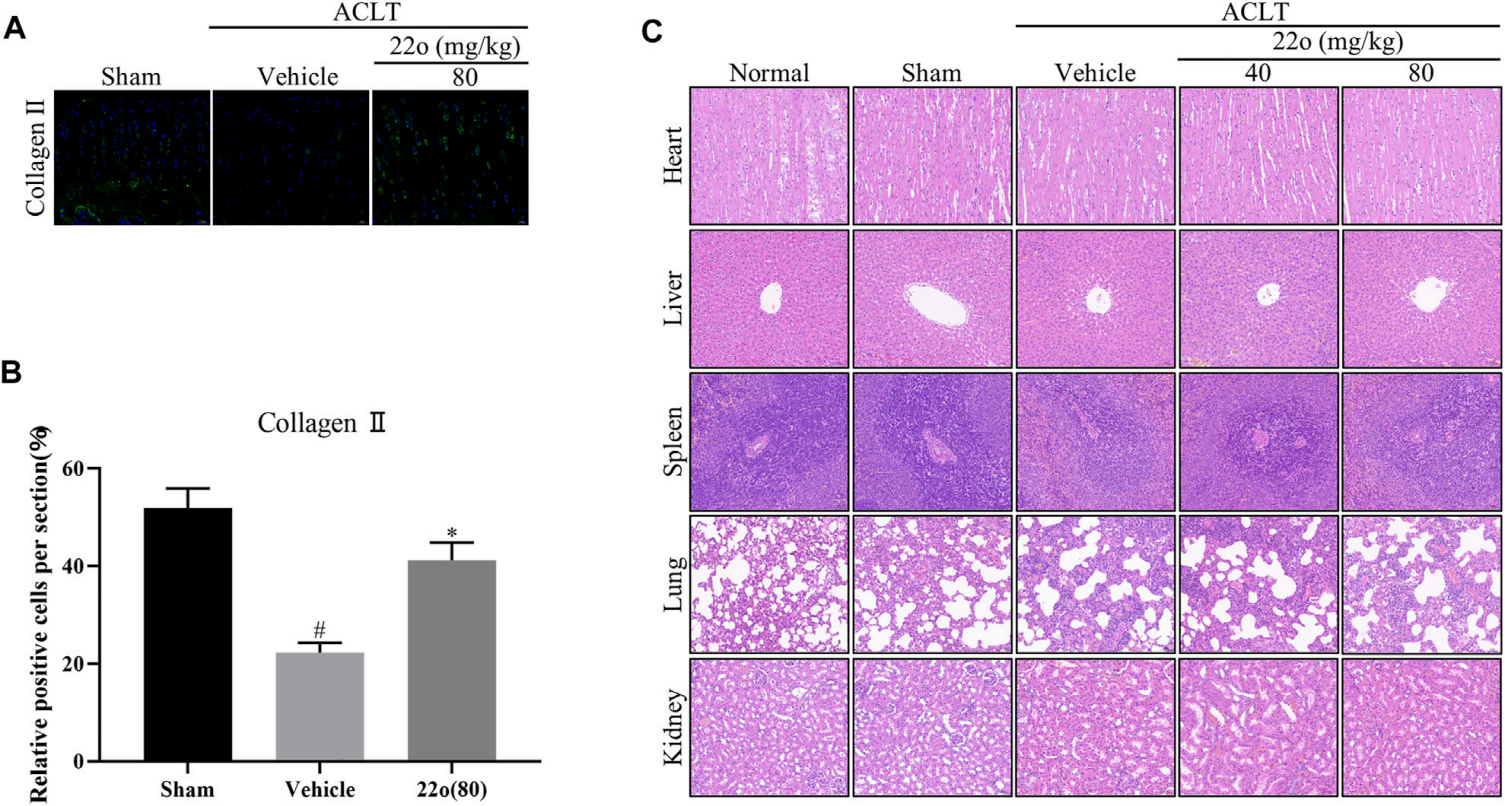
Compound 22o alleviates the progression of OA. **(A)** Representative images of immunofluorescent staining and **(B)** quantitative analysis of COL2A1 in the cartilage samples from three groups 6 weeks after treatment (scale bars, 20 μm, *n* = 6). **(C)** Representative images of HE staining of rat heart, liver, kidney, lung, and spleen from five groups 6 weeks after treatment (scale bars, 50 μm). #*p* < 0.05 vs. sham group; *p < 0.05 vs. vehicle group.

Next, the effect of compound 22o on rat OA was investigated *in vivo*. The experimental design for establishing the rat OA model via ACLT is shown in Figure 5A. The vehicle group showed apparent signs of cartilage injury, such as extensive cartilage erosion, proteoglycan loss, and chondrocyte disintegration (Figure 5B). However, compound 22o significantly inhibited the erosion of the OA articular cartilage and inhibited the loss of proteoglycans. The cartilage in the compound 22o treatment group exhibited a smoother and more complete structure than vehicle group. Immunohistochemical analysis showed that compound 22o significantly increased the expression of ACAN and COL2A1 and decreased the expression of MMP3 and MMP13 in the OA cartilage, which is consistent with the results of the *in vitro* experiments (Figures 5C,D). This was further confirmed by immunofluorescence staining with COL2A1; the level of COL2A1 in the compound 22o treatment group was significantly increased compared to that in the vehicle group (Figures 6A,B).

To evaluate the toxicity of compound 22o in the rats, the anatomical characteristics and HE staining of the heart, liver, spleen, lung, and kidney of the rats were examined. The anatomical structures of the heart, liver, spleen, lung, and kidney of the OA rats treated with compound 22o for 6 weeks appeared normal (Figure 6C), indicating that the compound was not toxic.

## Discussion

It is a fact that OA is a complex degenerative joint disease with an etiology involving age, obesity, joint trauma, chronic inflammation, and heredity (Creamer and Hochberg, 1997). However, the current therapeutic drugs used for OA can only alleviate the symptoms; no satisfactory DMOADs are available to date. In our previous study, we demonstrated the therapeutic effect of compound 22o on Freund’s adjuvant-induced rheumatoid arthritis (Chen et al., 2021), but its effect on OA remains unclear. Therefore, this study aimed to evaluate the anti-inflammatory and protective effect of the compound 22o on chondrocytes and the articular cartilage, respectively, in rats with OA. Compound 22o inhibited the activity of iNOS by inhibiting its dimerization, thereby reducing the production of NO and protecting the chondrocytes. Our results showed that compound 22o inhibited the inflammatory response and promoted the synthesis of cartilage ECM in the OA chondrocytes and the rat OA model, thus introducing a new potential candidate DMOAD that targets iNOS.

Various DMOADs have been reported in experimental and clinical studies for OA progression thus far. An experimental study found that BNTA could reverse the progression of OA by activating SOD3 and promoting the synthesis of cartilage ECM (Shi et al., 2019). Additionally, methylene blue can improve oxidative stress by regulating Nrf2/PRDX1 to prevent OA progression and relieve pain (Li et al., 2021). In other clinical studies, Sprifermin (Eckstein et al., 2021), TissueGene-C (Kim et al., 2018), and Lorecivivint (Yazici et al., 2020) demonstrated the potential to slow down the progression of OA. Nevertheless, these compounds have not yet been approved and are not available on the market. In this study, we chemically synthesized an iNOS dimerization inhibitor (compound 22o) that alleviated the progression of OA by regulating OA chondrocyte inflammation and ECM synthesis.

A local inflammatory response is vital for the occurrence and development of OA. In OA, many pro-inflammatory cytokines, such as IL-1β, TNF-α, and NO, are secreted in the joints. At the physiological level, NO, a key inflammatory mediator, is involved in the host defense function (Gantner et al., 2020). However, excessive NO can lead to abnormalities in the nervous system (Liu et al., 2015), vascular system (Tejero et al., 2019), gastrointestinal tract (Petersson et al., 2007), and immune system (Bogdan, 2011). Excess NO in OA not only inhibits the synthesis of cartilage ECM (Taskiran et al., 1994) and induces the expression of MMPs (Sasaki et al., 1998; Lepetsos and Papavassiliou, 2016) but also leads to chondrocyte apoptosis (Wu et al., 2007) and joint pain (Hancock and Riegger-Krugh, 2008). Therefore, controlling the amount of NO is particularly important for the occurrence and development of OA. The key to achieving this goal is to understand the regulatory mechanism of NO in chondrocytes and explore a new method to target it specifically.

NO is an important cell signal molecule produced by the NOS family via the oxidation of L-arginine to L-citrulline (Cinelli et al., 2020). In chondrocytes, NO is primarily induced by iNOS (Cheng et al., 2011). Abnormally increased pro-inflammatory cytokines induce the expression of iNOS and produce large amounts of NO, aggravating the pathological process of OA. Therefore, iNOS is a potential target for the treatment of OA. In this study, compound 22o significantly inhibited the activity of iNOS, thus reducing the production of NO as a result of compound 22o inhibiting the formation of dimer in the active form of iNOS. Our results show that compound 22o significantly inhibited the erosion of the OA articular cartilage and inhibited the loss of proteoglycans. The cartilage in the compound 22o treatment group exhibited a smoother and more complete structure than the vehicle group. Moreover, compound 22o alleviates the progression of OA. We believe that compound 22o can effectively protect the damaged joints in OA.

The iNOS inhibitors are a class of small molecules used to develop DMOADs. The iNOS inhibitors L-NMMA, L-NIL, and 1400W have been found to exert protective effects on the cartilage; however, the mechanisms have not yet been confirmed in clinical trials (Ahmad et al., 2020). Furthermore, almost all the existing NOS dimerization inhibitors contain imidazole moiety that interferes with the function of P450 enzymes resulting in some side effects. Compound 22o is a non-imidazole-based iNOS dimerization inhibitor. The iNOS dimerization inhibitor 22o showed a promising protective effect on the cartilage both *in vitro* and *in vivo* in the present study. In our previous study, compound 22o was found to have good drug-like properties and a high oral bioavailability; additionally, the acute toxicity and subacute toxicity tests revealed the safety of the compound *in vivo* (Chen et al., 2021). Further long-term toxicity tests are being conducted to provide a basis for subsequent clinical trials.

There are certain limitations to our study. The *in vitro* model involving IL-1β-induced chondrocytes is widely used, although it does not fully recapitulate the OA disease characteristics *in vivo*. Furthermore, chondrocytes are prone to dedifferentiation into fibroblasts after a small amount of passage, and both time- and concentration-dependent effects are also different. In addition, in the *in vivo* model of OA, we ignored the detection of circulating inflammatory markers.

## Conclusion

In conclusion, an iNOS dimerization inhibitor 22o was designed and synthesized in this study. It was found to inhibit the activity of iNOS and reduce the production of NO by inhibiting the formation of iNOS dimers, thus regulating OA chondrocyte inflammation and ECM synthesis. Considering the limitations associated with preclinical studies, subsequent clinical and experimental studies will be conducted to further support compound 22o as a DMOAD for the treatment of OA.

## Data Availability

The raw data supporting the conclusions of this article will be made available by the authors, without undue reservation.
